# Association Study of Two Cannabinoid Receptor Genes, CNR1 and CNR2, with Methamphetamine Dependence

**DOI:** 10.2174/157015911795017191

**Published:** 2011-03

**Authors:** Y Okahisa, M Kodama, M Takaki, T Inada, N Uchimura, M Yamada, N Iwata, M Iyo, I Sora, N Ozaki, H Ujike

**Affiliations:** 1 Department of Neuropsychiatry, Okayama University Graduate School of Medicine, Dentistry and Pharmaceutical Sciences, Okayama, Japan; 2 JGIDA (Japanese Genetics Initiative for Drug Abuse), Japan; 3 Seiwa Hospital, Tokyo, Japan; 4 Department of Neuropsychiatry, Kurume University Graduate School of Medicine, Kurume, Japan; 5 Department of Psychogeriatrics, National Institute of Mental Health, National Center of Neurology and Psychiatry, Kodaira, Japan; 6 Department of Psychiatry, Fujita Health University School of Medicine, Houmei, Japan; 7 Department of Psychiatry, Chiba University Graduate School of Medicine, Chiba, Japan; 8 Department of Neuroscience, Division of Psychobiology, Tohoku University Graduate School of Medicine, Sendai, Japan; 9 Department of Psychiatry, Nagoya University Graduate School of Medicine, Nagoya, Japan

**Keywords:** Substance abuse, methamphetamine, cannabinoid receptor 1, cannabinoid receptor 2, case-control association.

## Abstract

Several studies have suggested that the endocannabinoid system plays significant roles in the vulnerability to psychiatric disorders including drug abuse. To examine the possible association of the CNR1 and CNR2 genes, which encode cannabinoid receptors CB1 and CB2, with methamphetamine dependence, we investigated three single nucleotide polymorphisms (SNPs) (rs806379, rs1535255, rs2023239) in intron 2 of the CNR1 gene and a nonsynonymous SNP, Q63R, in the CNR2 gene. The study samples consisted of 223 patients with methamphetamine dependence and 292 age- and sex- matched controls. There were no significant differences between the patients and controls in genotypic or allelic distribution of any SNP of the CNR1 and CNR2 genes. We also analyzed the clinical features of methamphetamine dependence. Rs806379 of the CNR1 gene showed a significant association with the phenotype of latency of psychosis after the first consumption of methamphetamine. Patients with the T allele or T-positive genotypes (T/T or A/T) may develop a rapid onset of psychosis after methamphetamine abuse. The present study suggests a possibility that genetic variants of the CNR1 gene may produce a liability to the complication of psychotic state after abuse of methamphetamine; however, our findings need to be confirmed by future replications.

## INTRODUCTION

The endocannabinoid system is involved in vulnerability to psychiatric disorders including substance abuse [1]. There are two well-characterized cannabinoid receptors (CNRs), CNR1/CB1 and CNR2/CB2, that mediate endocannabinoid signaling [2]. CNR1 is distributed widely throughout the brain regions important for drug reward and drug memories, including the hippocampus, striatum, and cerebral cortex [3-6]. In animal studies, it was suggested that modulation of endogenous central cannabinoid signaling in the mesolimbic pathways may be one component of the addiction process, particularly *via* interaction with opioid and dopaminergic systems. *CNR1* knock-out mice display alterations in reward and drug-seeking behaviors in response to several substances, including nicotine [7, 8], ethanol [9, 10], amphetamine, cocaine, and other psychostimulants [11], and similar results were obtained after the blockade of CNR1 with the selective CNR1 antagonist, SR141716A [5, 12]. Some clinical studies have suggested that genetic variants of the CNR1 gene might be associated with susceptibility to substance dependence [13-19]. The polymorphism of the CNR1 genethat has been most widely studied is the (AAT)n trinucleotide short-tandem repeat. Comings *et al.* firstly reported that long (AAT)n repeats were associated with intravenous administration of drugs of abuse [15], but the result has not been confirmed by other studies [20-23]. Recently, Zhang *et al.* reported that the ‘TAG’ haplotype consisting of rs806379, rs1535255, and rs2023239 in the 5’ region of the CNR1 gene was related to polysubstance abuse in both European-Americans and African-Americans and also to alcohol dependence in Japanese [24]. They also showed that individuals carrying one copy of the ‘TAG’ haplotype showed lower levels of mRNA expression in the brain than non-carriers.

On the other hand, CNR2 has been traditionally referred to as the peripheral cannabinoid receptor because CNR2 is expressed mainly in some peripheral and immune cells [25-27]. However, recent studies demonstrated that CNR2 is also expressed in some regions of the brain and is supposed to be associated with addiction vulnerability as a modulator of the reward system [28-31]. A genetic variant of the CNR2 gene, Q63R, has been reported to be associated with autoimmune diseases [32] and osteoporosis [33]. Recently, Ishiguro *et al.* demonstrated that the variant is also associated with alcoholism in Japanese [34] and depression [35]. These findings suggest that CNR1 and CNR2 may be involved in substance abuse and endogenous psychosis. Based on the above rationale, we investigated the association between the CNR1 and CNR2 genes and methamphetamine dependence and psychosis in a Japanese population.

## METHODS

### Subjects

The subjects comprised 224 unrelated patients with methamphetamine dependence (178 males and 46 females, average age 37.0 ± 11.9 years) who met the ICD-10-DCR criteria (F15.2). Two hundred and six of these patients have or had comorbid methamphetamine psychosis (F15.5). They were in- or outpatients of psychiatric hospitals of the Japanese Genetics Initiative for Drug Abuse (JGIDA). The healthy controls were 292 normal, unrelated, age-, sex-, and geographical origin-matched individuals (228 males and 64 females, average age 37.2 ± 13.1 years). Most were medical staff members who had no past history or family history of substance dependence or major psychotic disorder. All subjects were Japanese, born and living in restricted areas of Japan, including northern Kyushu, Setouchi, Chukyo, Tokai, and Kanto, regions whose genetic background is considered to represent relative unity. This study was initiated after receiving the approval of the ethical committees of the participating institutions of the JGIDA. Written informed consent was obtained from all participants.

### Clinical Phenotypes

Clinical observation has revealed substantial inter-individual differences in certain phenotypes of methamphetamine-taking behavior and psychosis that seem to be regulated, at least in part, genetically, and the rationale and methods of the subgrouping were previously described [36]. In brief, the patients with methamphetamine dependence and psychosis were divided into five subgroups according to the following clinical phenotypes: multisubstance-abuse status, age at first consumption of methamphetamine, latency to the onset of psychotic symptoms after the first consumption of methamphetamine, prognosis of psychosis after therapy, and the complication of spontaneous relapse to a psychotic state.

### Genotyping

Peripheral blood was obtained from the subjects, and the genomic DNA was extracted from peripheral leukocytes using a standard procedure. We selected four SNPs, rs806379, rs1535255 and rs2023239, of the CNR1 gene and a nonsynonymous polymorphism, Q63R, of the CNR2 gene, for genetic association analyses. The three SNPs of the CNR1 gene are located in the distal region of intron 2 and were reported to be associated with polysubstance abuse by Zhang *et al.* [39]. Genotyping of the three SNPs of the CNR1 gene was performed using TaqMan technology on an ABI7500 Real Time PCR system (Applied Biosystems, U.S.A.). The genotypes of Q63R of the CNR2 gene were determined by the polymerase chain reaction (PCR)-restriction fragment length polymorphism (RFLP) method as previously reported [34]. All genotyping was performed in a blinded fashion, with the control and case samples mixed randomly.

### Statistical Analysis

Statistical analysis of association was performed using SNPAlyze software (Dynacom Co., Japan). Deviation from Hardy-Weinberg equilibrium and the case-control study were tested using the χ^2 ^test. Linkage disequilibrium (LD) was tested using the χ^2 ^test, and *D’* and *r*^2^ values were made the index in the authorization of LD. Case-control haplotype analysis was performed by the permutation method, and permutation *p* values were calculated based on 100,000 replications.

## RESULTS

The genotype distribution and allele frequencies for each polymorphism of patients with methamphetamine dependence and control subjects are shown in Table **1**. The genotype distributions of patients with methamphetamine dependence and control subjects did not deviate from Hardy-Weinberg equilibrium at any of the four SNPs. We found no significant differences in genotypic or allelic distribution at any polymorphism of the CNR1 or CNR2 gene between the patients and controls.

We estimated the pairwise LD between the three SNPs of the CNR1 gene using the D’ and r^2^ values as an index, and we found that rs806379, rs1535255, and rs2023239 showed a strong LD (D’ ranging from 0.90 to 0.99) with each other (Table **2**). We then analyzed the 2- and 3-loci haplotype distribution, but no significant difference was found between patients of methamphetamine dependence and control subjects (global permutation *p* value of 3-loci haplotype was 0.34, Tables **3** and **4**).

To investigate further the roles of CNR1 and CNR2 in the pathophysiology of psychosis and drug-taking behaviors, we examined the association of CNR1 and CNR2 genes with several clinical phenotypes of methamphetamine dependence and psychosis, such as the age at first consumption of methamphetamine, latency to onset of psychosis after abuse, prognosis of psychosis after therapy, spontaneous relapse even without reconsumption of methamphetamine, and multiple substance abuse status, which show individual variation and may in part be regulated genetically. There was a significant difference in the allelic distribution of rs806379 of the CNR1 gene between the two subgroups divided by latency to onset of psychosis after methamphetamine abuse (Table **5**). The T allele, a minor allele of rs806379, was more found frequently in the patients with a shorter latency to onset of psychosis than those with a longer latency (P=0.040). The patients with T-positive genotypes, A/T and T/T, also showed a shorter latency to onset of psychosis than those without (P=0.039). The other SNPs, rs1535255 and rs2023239 of the CNR1 and Q63R of the CNR2 gene, did not show an association with either phenotype (Table **5**).

## DISCUSSION

The present study showed that the CNR1 and CNR2 genes were not associated with methamphetamine dependence/psychosis, but that the CNR1 gene was associated with one clinical phenotype: latency to the onset of psychotic symptoms after the first consumption of methamphetamine. Individuals with the T allele or T-positive genotypes of rs rs806379 showed a rapid onset of psychosis after methamphetamine abuse. This finding suggests that the CNR1 gene may contribute to a liability for the complication of psychotic symptoms after psychostimulant abuse.

Zhang *et al.* [24] reported that the ‘TAG’ haplotype consisting of rs806379, rs1535255, and rs2023239 of the CNR1 gene, the three SNPs of the CNR1 gene that we genotyped in the present study, were strongly associated with polysubstance abuse both in European- and African-Americans. Additionally, they identified the ‘TAG’ haplotype in Japanese and found a significant association with alcoholism in Japanese. To identify the functional effects of the ‘TAG’ haplotype, they measured the expression levels of *CNR1* mRNAs and found striking differences in the *CNR1* mRNA levels in the brains of those with the ‘TAG’ haplotype and the other haplotypes. In recent years, several studies have examined the associations of the CNR1 gene with substance abuse and dependence, but the results were not always consistent [13, 14, 16-23, 37-40]. Our data indicated that the polymorphisms of the CNR1 gene affected the clinical course of methamphetamine psychosis, but not dependence, at least in a Japanese population.

With regard to the CNR2 gene, Ishiguro *et al.* reported that the nonsynonymous polymorphism, Q63R, of the CNR2 gene was associated with alcoholism in a Japanese population [34], and they showed an association between the polymorphism and Japanese patients of depression in another study [35]. They also revealed that mice expressing alcohol preference showed reduced Cb2 gene expression in the brain [34]. Furthermore, a significant decrease of the levels of the CB2 mRNA has been reported to accompany clinical remission of schizophrenia [41]. However, our data indicate that the polymorphism of the CNR2 gene did not affect the risk of methamphetamine dependence and psychosis or the clinical phenotypes of methamphetamine psychosis in a Japanese population.

Several concerns about the present study should be raised. We found a statistically significant association of the rs806379 of the CNR1 gene with one of the clinical phenotypes, latency to the onset of psychotic symptoms after the first consumption of methamphetamine, but the *p* value was marginal. The possibility of type I errors should be considered. Further, we examined these three SNPs of the CNR1 gene and one SNP of the CNR2 gene because they may have physiological effects, but there are many other SNPs in the CNR1 and CNR2 genes. Therefore, our findings must be confirmed in larger samples by examining additional SNPs to cover the entire CNR1 and CNR2 genes. Finally, the minor allele frequencies of the three SNPs of the CNR1 gene shown in Table **1** are markedly different from those reported by Zhang *et al.* [24]. The differences may be due to population differences or a sample size not large enough for comparison. We reported the SNPs based on the NCBI refSNP marker database, which corresponds to the chromosome 6-plus strand in each case. Zhang *et al.* have may used the base designation for the chromosome 6-minus strand for these markers. Accordingly, the ‘TAG’ haplotype reported by Zhang *et al.* corresponded to the ‘ATC’ haplotype of our study. However, the ‘ATC’ haplotype was completely absent in our population.

In conclusion, this study showed that *CNR1* variation may contribute to liability for complications of psychotic symptoms of methamphetamine abusers. Further research is needed to determine the possible association between endocannabinoid system and substance dependence.

## Figures and Tables

**Table 1:** Genotype and Allele Frequencies of Three Single Nucleotide Polymorphisms of the CNR1 Gene and one SNP of the CNR2 Gene in Patients with METH Dependence and/or Psychosis and Controls

		Genotype(%)			*P*	Allele(%)		*P*
CNR1 rs806379	N	A/A	A/T	T/T		A	T	
Controls	290	186(64.2)	92(31.7)	12(4.1)	0.49	464(80.0)	116(20.0)	0.23
Patients	223	154(69.1)	62(27.8)	7(3.1)		370(83.0)	76(17.0)	
CNR1 rs1535255	N	T/T	T/G	G/G		T	G	
Controls	292	267(91.4)	25(85.6)	0(0)	0.87	559(95.7)	25(4.3)	0.87
Patients	223	203(91.0)	20(9.0)	0(0)		426(95.5)	20(4.5)	
CNR1 rs2023239	N	T/T	T/C	C/C		T	C	
Controls	292	266(91.1)	26(8.9)	0(0)	0.97	558(95.5)	26(4.5)	0.97
Patients	222	202(91.0)	20(9.0)	0(0)		424(95.5)	20(4.5)	
CNR2 Q63R	N	gg/gg(R/R)	gg/aa(R/Q)	aa/aa(Q/Q)		gg(Arg)	aa(Glu)	
Controls	281	93(33.1)	134(47.7)	54(19.2)	0.97	320(56.9)	242(43.1)	0.95
Patients	223	72(32.3)	109(48.9)	42(18.8)		253(56.7)	193(43.3)	

**Table 2:** Liskage Disequilibrium Analyses of three SNPs of the CNR1 Gene

	rs806389	rs1535255	rs2023239

rs806389		0.97	0.90
rs1535255	0.19		0.98
rs2023239	0.17	0.93	

Linkage disequilibrium (LD) was tested using χ^2^ test.

Right upper and left lower diagonal showed D' and r-square values, respectively.

**Table 3:** Multi-Loci Association Analyses of the CNR1 Gene

SNP	1 Locus	2 Loci	3 Loci

rs806379	0.23		
		0.36	
rs1535255	0.87		0.34
		0.56	
rs2023239	0.97		

**Table 4:** Haplotype Frequencies of the CNR1 Gene

	rs806379/rs1535255/rs2023239		
Haplotype	Control	Patient	Permutation p-value
A-T-T	0.7944	0.8288	0.15
T-T-T	0.159	0.1261	0.14
T-G-C	0.0393	0.045	0.64

Global permutation p-value = 0.34.

**Table 5:** Subgroups of METH Dependence and Psychosis by Clinical Characteristic

Clinical Phenotype		Genotype(%)			*P*	Allele(%)		*P*
CNR1 rs806379	N	A/A	A/T	T/T		A	T	
Multisubstance abuse								
Yes	157	109(69.4)	42(26.8)	6(3.8)	0.66	260(82.8)	54(17.2)	0.95
No	59	40(67.8)	18(30.5)	1(1.7)		98(83.1)	20(16.9)	
Age at first consumption								
≤20	118	79(66.9)	37(31.4)	2(1.7)	0.21	195(82.6)	41(17.4)	0.84
>20	102	73(71.6)	24(23.5)	5(4.9)		170(83.3)	34(16.7)	
Latency to onset of psychosis								
≤3 years	102	64(62.8)	34(33.3)	4(3.9)	0.12	162(79.4)	42(20.6)	0.04
>3 years	93	71(76.3)	20(21.5)	2(2.2)		162(87.1)	24(12.9)	
Prognosis of psychosis								
Transient	113	76(67.3)	33(29.2)	4(3.5)	0.81	185(81.9)	41(18.1)	0.56
Prolonged	91	65(71.4)	23(25.3)	3(3.3)		153(84.1)	29(15.9)	
Spontaneous relapse of psychosis								
Yes	93	69(74.2)	22(23.7)	2(2.1)	0.41	160(86.0)	26(14.0)	0.18
No	119	79(66.4)	35(29.4)	5(4.2)		193(81.1)	45(18.9)	
CNR1 rs1535255	N	T/T	T/G	G/G		T	G	
Multisubstance abuse								
Yes	157	143(91.1)	14(8.9)	0(0.0)	0.78	300(95.5)	14(4.5)	0.78
No	59	53(89.8)	6(10.2)	0(0.0)		112(94.9)	6(5.1)	
Age at first consumption								
≤20	118	108(91.5)	10(8.5)	0(0.0)	0.93	226(95.8)	10(4.2)	0.93
>20	102	93(91.2)	9(8.8)	0(0.0)		195(95.6)	9(4.4)	
Latency to onset of psychosis								
≤3 years	102	91(89.2)	11(10.8)	0(0.0)	0.61	193(94.6)	11(5.4)	0.62
>3 years	93	85(91.4)	8(8.6)	0(0.0)		178(95.7)	8(4.3)	
Prognosis of psychosis								
Transient	113	101(89.4)	12(10.6)	0(0.0)	0.47	214(94.7)	12(5.3)	0.49
Prolonged	91	84(92.3)	7(7.7)	0(0.0)		175(96.2)	7(3.8)	
Spontaneous relapse of psychosis								
Yes	93	84(90.3)	9(9.7)	0(0.0)	0.58	177(95.2)	9(4.8)	0.59
No	119	110(92.4)	9(7.6)	0(0.0)		229(96.2)	9(3.8)	
CNR1 rs2023239	N	T/T	T/C	C/C		T	C	
Multisubstance abuse								
Yes	156	142(91.0)	14(9.0)	0(0.0)	0.79	298(95.5)	14(4.5)	0.79
No	59	53(89.8)	6(10.2)	0(0.0)		112(94.9)	6(5.1)	
Age at first consumption								
≤20	117	107(91.5)	10(8.5)	0(0.0)	0.94	224(95.7)	10(4.3)	0.94
>20	102	93(91.2)	9(8.8)	0(0.0)		195(95.6)	9(4.4)	
Latency to onset of psychosis								
≤3 years	102	91(89.2)	11(10.8)	0(0.0)	0.61	193(94.6)	11(5.4)	0.62
>3 years	93	85(91.4)	8(8.6)	0(0.0)		178(95.7)	8(4.3)	
Prognosis of psychosis								
Transient	113	101(89.4)	12(10.6)	0(0.0)	0.49	214(94.7)	12(5.3)	0.50
Prolonged	90	83(92.2)	7(7.8)	0(0.0)		173(96.1)	7(3.9)	
Spontaneous relapse of psychosis								
Yes	92	83(90.2)	9(9.8)	0(0.0)	0.57	175(95.1)	9(4.9)	0.58
No	119	110(92.4)	9(7.6)	0(0.0)		229(96.2)	9(3.8)	
CNR2 Q63R	N	gg/gg(R/R)	gg/aa(R/Q)	aa/aa(Q/Q)		gg(Arg)	aa(Glu)	
Multisubstance abuse								
Yes	157	53(33.8)	74(47.1)	30(19.1)	0.64	180(57.3)	134(42.7)	0.46
No	59	16(27.1)	31(52.5)	12(20.4)		63(53.4)	55(46.6)	
Age at first consumption								
≤20	119	37(31.1)	58(48.7)	24(20.2)	0.81	132(55.5)	106(44.5)	0.52
>20	100	34(34.0)	49(49.0)	17(17.0)		117(58.5)	83(41.5)	
Latency to onset of psychosis								
≤3 years	102	33(32.4)	55(53.9)	14(13.7)	0.08	121(59.3)	83(40.7)	0.19
>3 years	92	29(31.5)	39(42.4)	24(26.1)		97(52.7)	87(47.3)	
Prognosis of psychosis								
Transient	112	40(35.7)	53(47.3)	19(17.0)	0.41	133(59.4)	91(40.6)	0.18
Prolonged	92	26(28.3)	45(48.9)	21(22.8)		97(52.7)	87(47.3)	
Spontaneous relapse of psychosis								
Yes	93	30(32.3)	43(46.2)	20(21.5)	0.69	103(55.4)	83(44.6)	0.84
No	118	36(30.5)	61(51.7)	21(17.8)		133(56.4)	103(43.6)	

## References

[1] van der Stelt M, Di Marzo V (2003). The endocannabinoid system in the basal ganglia and in the mesolimbic reward system: implica-tions for neurological and psychiatric disorders. Eur. J. Pharmacol.

[2] Matsuda LA, Lolait SJ, Brownstein MJ, Young AC, Bonner TI (1990). Structure of a cannabinoid receptor and functional expression of the cloned cDNA. Nature.

[3] Herkenham M, Lynn AB, Johnson MR, Melvin LS, de Costa BR, Rice KC (1991). Characterization and localization of cannabinoid receptors in rat brain: a quantitative *in vitro* autoradiographic study. J. Neurosci.

[4] Herkenham M, Lynn AB, Little MD, Johnson MR,  
Melvin LS, de Costa BR, Rice KC (1990). Cannabinoid receptor localization in brain. Proc. Natl. Acad. Sci. USA.

[5] Matsuda LA, Bonner TI, Lolait SJ (1993). Localization of cannabinoid receptor mRNA in rat brain. J. Comp. Neurol.

[6] Tsou K, Brown S, Sanudo-Pena MC, Mackie K, Walker JM (1998). Immunohistochemical distribution of cannabinoid CB1 receptors in the rat central nervous system. Neuroscience.

[7] De Vries TJ, Schoffelmeer AN (2005). Cannabinoid CB1 receptors control conditioned drug seeking. Trends Pharmacol. Sci.

[8] Forget B, Hamon M, Thiebot MH (2005). Cannabinoid CB1 receptors are involved in motivational effects of nicotine in rats. Psychopharmacology (Berl).

[9] Colombo G, Serra S, Vacca G, Carai MA, Gessa GL (2005). Endocannabinoid system and alcohol addiction: pharmacological studies. Pharmacol. Biochem. Behav.

[10] Racz I, Bilkei-Gorzo A, Toth ZE, Michel K, Palkovits M, Zimmer A (2003). A critical role for the cannabinoid CB1 receptors in alcohol dependence and stress-stimulated ethanol drinking. J. Neurosci.

[11] Maldonado R, Valverde O, Berrendero F (2006). Involvement of the endocannabinoid system in drug addiction. Trends Neurosci.

[12] Yamamoto T, Takada K (2000). Role of cannabinoid receptor in the brain as it relates to drug reward. Jpn. J. Pharmacol.

[13] Agrawal A, Wetherill L, Dick DM, Xuei X, Hinrichs A, Hesselbrock V, Kramer J, Nurnberger JI Jr, Schuckit M, Bierut LJ, Edenberg HJ, Foroud T (2008). Evidence for association between polymorphisms in the cannabinoid receptor 1 (CNR1) gene and cannabis dependence. Am. J. Med. Genet. B Neuropsychiatr. Genet.

[14] Chen X, Williamson VS, An SS, Hettema JM, Aggen SH, Neale MC, Kendler KS (2008). Cannabinoid receptor 1 gene association with nicotine dependence. Arch. Gen. Psychiatry.

[15] Comings DE, Muhleman D, Gade R, Johnson P, Verde  
R, Saucier G, MacMurray J (1997). Cannabinoid receptor gene (CNR1): association with i.v. drug use. Mol. Psychiatry.

[16] Herman AI, Kranzler HR, Cubells JF, Gelernter J, Covault J (2006). Association study of the CNR1 gene exon 3 alternative promoter region polymorphisms and substance dependence. Am. J. Med. Ge-net. B Neuropsychiatr. Genet.

[17] Hopfer CJ, Young SE, Purcell S, Crowley TJ, Stallings MC, Corley RP, Rhee SH, Smolen A, Krauter K, Hewitt JK, Ehringer MA (2006). Cannabis receptor haplotype associated with fewer cannabis dependence symptoms in adolescents. Am. J. Med. Genet. B Neuropsychiatr. Genet.

[18] Schmidt LG, Samochowiec J, Finckh U, Fiszer-Piosik E, Horodnicki J, Wendel B, Rommelspacher H, Hoehe MR (2002). Association of a CB1 cannabinoid receptor gene (CNR1) polymorphism with severe alcohol dependence. Drug Alcohol Depend.

[19] Zuo L, Kranzler HR, Luo X, Covault J, Gelernter J (2007). CNR1 variation modulates risk for drug and alcohol dependence. Biol. Psychiatry.

[20] Ballon N, Leroy S, Roy C, Bourdel MC, Charles-Nicolas A, Krebs MO, Poirier MF (2006). (AAT)n repeat in the cannabinoid  receptor gene (CNR1): association with cocaine addiction in  an African-Caribbean population. Pharmacogenomics J.

[21] Covault J, Gelernter J, Kranzler H (2001). Association study of  cannabinoid receptor gene (CNR1) alleles and drug dependence. Mol. Psychiatry.

[22] Li T, Liu X, Zhu ZH, Zhao J, Hu X, Ball DM, Sham PC, Collier DA (2000). No association between (AAT)n repeats in the cannabinoid receptor gene (CNR1) and heroin abuse in a Chinese population. Mol. Psychiatry.

[23] Ponce G, Hoenicka J, Rubio G, Ampuero I, Jimenez-Arriero MA, Rodriguez-Jimenez R, Palomo T, Ramos JA (2003). Association between cannabinoid receptor gene (CNR1) and childhood attention deficit/hyperactivity disorder in Spanish male alcoholic patients. Mol. Psychiatry.

[24] Zhang PW, Ishiguro H, Ohtsuki T, Hess J, Carillo F,  
Walther D, Onaivi ES, Arinami T, Uhl GR (2004). Human 
cannabinoid receptor 1: 5' exons, candidate regulatory regions, polymorphisms, haplotypes and association with polysubstance abuse. Mol. Psychiatry.

[25] Berdyshev EV (2000). Cannabinoid receptors and the regulation of immune response. Chem. Phys. Lipids.

[26] Munro S, Thomas KL, Abu-Shaar M (1993). Molecular characterization of a peripheral receptor for cannabinoids. Nature.

[27] Sugiura T, Waku K (2002). 2-Arachidonoylglycerol and the cannabinoid receptors. Chem. Phys. Lipids.

[28] Onaivi ES, Carpio O, Ishiguro H, Schanz N, Uhl GR, Benno R (2008). Behavioral effects of CB2 cannabinoid receptor activation and its influence on food and alcohol consumption. Ann. N. Y. Acad. Sci.

[29] Onaivi ES, Ishiguro H, Gong JP, Patel S, Meozzi PA, Myers L, Perchuk A, Mora Z, Tagliaferro PA, Gardner E, Brusco A, Akinshola BE, Hope B, Lujilde J, Inada T, Iwasaki S, Macharia D, Teasenfitz L, Arinami T, Uhl GR (2008). Brain neuronal CB2 cannabinoid receptors in drug abuse and depression: from mice to human subjects. PLoS ONE.

[30] Onaivi ES, Ishiguro H, Gong JP, Patel S, Perchuk A, Meozzi PA, Myers L, Mora Z, Tagliaferro P, Gardner E, Brusco A, Akinshola BE, Liu QR, Hope B, Iwasaki S, Arinami T, Teasenfitz L, Uhl GR (2006). Discovery of the presence and functional expression of cannabinoid CB2 receptors in brain. Ann. N. Y. Acad. Sci.

[31] Van Sickle MD, Duncan M, Kingsley PJ, Mouihate A,  
Urbani P, Mackie K, Stella N, Makriyannis A, Piomelli D, Davison JS, Marnett LJ, Di Marzo V, Pittman QJ, Patel KD, Sharkey KA (2005). Identification and functional characterization of brainstem cannabinoid CB2 receptors. Science.

[32] Sipe JC, Arbour N, Gerber A, Beutler E (2005). Reduced endocannabinoid immune modulation by a common cannabinoid 2 (CB2) receptor gene polymorphism: possible risk for autoimmune disorders. J. Leukoc. Biol.

[33] Karsak M, Cohen-Solal M, Freudenberg J, Ostertag A, Morieux C, Kornak U, Essig J, Erxlebe E, Bab I, Kubisch C, de Vernejoul MC, Zimmer A (2005). Cannabinoid receptor type 2 gene is associated with human osteoporosis. Hum. Mol. Genet.

[34] Ishiguro H, Iwasaki S, Teasenfitz L, Higuchi S, Horiuchi Y, Saito T, Arinami T, Onaivi ES (2007). Involvement of cannabinoid CB2 receptor in alcohol preference in mice and alcoholism in humans. Pharmacogenomics J.

[35] Onaivi ES, Ishiguro H, Gong JP, Patel S, Meozzi PA, Myers L, Perchuk A, Mora Z, Tagliaferro PA, Gardner E, Brusco A, Akinshola BE, Liu QR, Chirwa SS, Hope B, Lujilde J, Inada T, Iwasaki S, Macharia D, Teasenfitz L, Arinami T, Uhl GR (2008). Functional expression of brain neuronal CB2 cannabinoid receptors are involved in the effects of drugs of abuse and in depression. Ann. N. Y. Acad. Sci.

[36] Ujike H, Sato M (2004). Clinical features of sensitization to methamphetamine observed in patients with methamphetamine dependence and psychosis. Ann. N. Y. Acad. Sci.

[37] Hartman CA, Hopfer CJ, Haberstick B, Rhee SH, Crowley TJ, Corley RP, Hewitt JK, Ehringer MA (2009). The association between cannabinoid receptor 1 gene (CNR1) and cannabis dependence symptoms in adolescents and young adults. Drug Alcohol Depend.

[38] Hoenicka J, Ponce G, Jimenez-Arriero MA, Ampuero I, Rodriguez-Jimenez R, Rubio G, Aragues M, Ramos JA, Pa-lomo T (2007). Association in alcoholic patients between psychopathic traits and the additive effect of allelic forms of the CNR1 and FAAH endocannabinoid genes, and the 3' region of the DRD2 gene. Neurotox. Res.

[39] Preuss UW, Koller G, Zill P, Bondy B, Soyka M (2003). Alcoholism-related phenotypes and genetic variants of the CB1 receptor. Eur. Arch. Psychiatry Clin. Neurosci.

[40] Zuo L, Kranzler HR, Luo X, Yang BZ, Weiss R, Brady K, Poling J, Farrer L, Gelernter J (2009). Interaction between two independent CNR1 variants increases risk for cocaine dependence in European Americans: a replication study in family-based sample and population-based sample. Neuropsychopharmacology.

[41] De Marchi N, De Petrocellis L, Orlando P, Daniele F, Fezza F, Di Marzo V (2003). Endocannabinoid signalling in the blood of patients with schizophrenia. Lipids Health Dis.

